# Effects of Kinesio Tape on Delayed Onset Muscle Soreness: A Systematic Review and Meta-analysis

**DOI:** 10.1155/2021/6692828

**Published:** 2021-05-31

**Authors:** Jianping Lin, Ming ling Guo, Hao Wang, Cheng Lin, Guiqing Xu, Aiping Chen, Shaoqing Chen, Shizhong Wang

**Affiliations:** ^1^School of Health, Fujian Medical University, Fuzhou, China; ^2^Center for Collaborative Innovation in Geriatric Rehabilitation and Industry Promotion, Fujian Medical University, Fuzhou, China; ^3^College of Rehabilitation Medicine, Fujian University of Traditional Chinese Medicine, Fuzhou, China

## Abstract

**Background:**

Kinesio tape (KT) may be useful for the treatment of delayed onset muscle soreness (DOMS), but there has been no systematic review assessing their efficacy.

**Objectives:**

We conducted a systematic review and meta-analysis to evaluate the efficacy of KT on DOMS.

**Methods:**

We searched seven databases for randomized controlled trials (RCTs) and crossover randomized trials of KT in DOMS, from the earliest date available to December 31, 2019. The primary outcome was muscle soreness. The secondary outcome was muscle strength and serum creatine kinase (CK) level. The risk of bias was evaluated based on the Cochrane criteria. Data were analyzed using RevMan version 5.3.0 software. *P* values < 0.05 were considered statistically significant. Systematic review registration number is CRD42020157052.

**Results:**

Eight trials (six RCTs and two crossover randomized trials) with 289 participants were included. KT use significantly reduced muscle soreness at 48 h (mean difference (MD): -0.67, 95% confidence interval (CI): -1.10 to 0.24, *P* = 0.002) and 72 h postexercise (MD: -0.81, 95% CI: -1.45 to -0.17, *P* = 0.01) but not at 24 h. KT use improved muscle strength at 72 h postexercise (standardized mean difference: 0.35, 95% CI: 0.02 to 0.69, *P* = 0.04) but not at 24 or 48 h. However, the serum CK level at 24, 48, and 72 h postexercise was not better in the KT group relative to the control group.

**Conclusions:**

Current evidence suggests that KT might help to alleviate DOMS after strenuous exercise to improve muscle strength. Thus, using KT on the skin for more than 48 hours postexercise, but not for 24 h, appears more effective at relieving pain and improving muscle strength.

## 1. Introduction

In recent years, more people worldwide have begun to take part in and advocate for exercise as an essential element of a healthy lifestyle. Exercise is beneficial for preventing and treating chronic diseases, such as metabolic (e.g., obesity, diabetes), cardiovascular (e.g., hypertension, coronary heart disease), and psychiatric (e.g., depression, anxiety, stress) diseases [1]. However, being unaccustomed to exercise or strenuous exercises, especially those involving eccentric muscle contractions, may cause negative effects, such as delayed onset muscle soreness (DOMS) [2]. DOMS is often accompanied by muscle soreness, stiffness, and dysfunction [3]. This muscle soreness occurs during the first 24 h after intense or exhaustive exercise and peaks from 24 to 72 h postexercise, persisting for 5 to 7 days before full recovery [4, 5]. Because of the reduction in joint range of motion and muscle strength, DOMS adversely affects physical performance during consecutive training or games [6]. And the reduction in joint range of motion and muscle strength and symptoms of DOMS impairs the quality of training [7, 8]. A variety of strategies have been implemented to prevent or reduce the symptoms of DOMS, including anti-inflammatory drug therapy, physical therapy, acupuncture, and massage. However, there is no standard treatment for DOMS.

Kinesio tape (KT), which was developed by a chiropractor named Kenzo Kase, is widely used for rehabilitation and prevention of sports injuries [9]. The potential effectiveness of KT is linked to its reported stimulation of cutaneous receptors, promotion of lymphatic and blood circulation, remodeling of the joints and fascia, restoration of muscle function by strengthening weak muscles, and inhibition of pain sensitivity by increasing touch and proprioceptive input [2, 9]. These mechanisms, particularly increased blood flow, may be beneficial for the elimination of muscle-specific protein following intense exercise and appear to reduce muscle inflammatory response, which was thought to bring about secondary damage to myofibrillar proteins [10]. Given these characteristics, it is possible that KT may be an effective tool for promoting recovery from DOMS.

Several studies have demonstrated the beneficial effects of KT, including pain reduction and improved muscle function and strength in multiple muscles in exercise-induced muscle damage (e.g., quadriceps femoris, biceps brachii, and gastrocnemius) [4, 11–13]. However, not all reports on KT have been positive. For instance, Ozmen et al. [9] reported that KT was applied immediately before the squat exercise and remained on the skin for 48 h, which did not relieve muscle pain or improve sprint performance 48 h postexercise. Fu et al. [14] and Vercelli et al. [15] similarly reported that KT did not improve quadriceps and hamstring muscle strength immediately or 12 h after taping in healthy noninjured subjects. Furthermore, de Almeida Lins et al. [16] found that KT did not change lower limb function, monopod static balance, or peak knee extensor muscle torque immediately after taping in healthy noninjured subjects. These findings suggest that the beneficial effects of KT on DOMS are controversial; therefore, a systematic review is needed to determine whether KT is effective in alleviating DOMS postexercise.

We hypothesize that KT is effective for treating DOMS induced by strenuous exercise. To test this hypothesis, we evaluated the available evidence to determine the effect of KT on DOMS using a systematic review and meta-analysis approach. To the best of our knowledge, this is the only systematic review and meta-analysis to formally assess whether KT is effective in alleviating DOMS at multiple time points postexercise.

## 2. Methods

### 2.1. Experimental Approach to the Problem

Four English (Cochrane Library, PubMed, the Physiotherapy Evidence Database (PEDro), and EMBASE) and three Chinese databases (China National Knowledge Information Database (CNKI), Chinese Scientific Journal Database (VIP), and Wang Fang) were searched. Original research articles with a publication date prior to December 31, 2019, were searched using the following keywords: “Kinesio Tape,” “delayed-onset muscle soreness,” “exercise,” and “muscle.” Further details on the search strategy are given in the Supplementary Data S1. Article languages were restricted to Chinese and English. The guidelines of the Preferred Reporting Items for Systematic Reviews and Meta-Analyses (PRISMA) were used to present detailed results (Supplementary Data S2) [17]. The PROSPERO registration number for this systematic review is CRD42020157052.

### 2.2. Inclusion Criteria

Clinical studies that met the following criteria were included: randomized controlled trials (RCTs) and crossover randomized trials in humans with a major aim of evaluating the effectiveness of KT for DOMS; KT as the intervention; control individuals who received placebo tape treatment, no treatment, or passive rest; outcomes measures were muscle soreness (pain or soreness rating), muscle strength (maximal isometric muscle strength or peak torque), or serum creatine kinase (CK) levels.

### 2.3. Exclusion Criteria

Clinical studies were excluded if they were only an abstract, reported no mean value (for the primary or secondary outcomes) or standard deviation (SD), and if neither could be determined even after contacting the author.

### 2.4. Study Selection

Two reviewers independently screened all study titles and abstracts. The full text of the studies that potentially met the inclusion criteria (based on title and abstract screening) was obtained, and all potentially relevant references were retrieved according to the predefined inclusion criteria. Differences were resolved by discussion or, if necessary, by consultation with a third investigator to reach a consensus.

### 2.5. Methodological Quality Assessment

To determine quality of the included studies, the risk of bias was evaluated based on the assessment criteria in the Cochrane Handbook for Systematic Review [17, 18], according to the following six domains: selection bias, performance bias, detection bias, attrition bias, reporting bias, and other bias. Each form of bias was classified as low, high, or unclear. Two reviewers independently judged each domain to assess whether there was a low, high, or an unclear bias. In cases of inconsistency between the two reviewers, a third reviewer was consulted, and consensus was reached by discussion.

### 2.6. Data Extraction

Two investigators participated in data extraction from all publications included in the present study. One investigator performed the initial data extraction and a second investigator subsequently reexamined each article and verified the results.

### 2.7. Statistical Analyses

Cochrane Collaboration Review Manager Software (RevMan version 5.3.0) was used for all statistical analyses. Outcomes assessed by RevMan (muscle soreness and CK) were assessed as mean difference (MD), whereas the standardized mean difference (SMD) was used to analyze effects when the same index was measured in different ways. Heterogeneity was assessed by calculating *I*^2^ values. *I*^2^ < 25% indicated low heterogeneity, 25%–75% indicated moderate heterogeneity, and ≥75% indicated high heterogeneity. When *I*^2^ was ≤25%, fixed-effects models were used. When *I*^2^ was >25%, a random-effects model was applied [17]. Fixed-effects models assume that the population effect sizes are the same for all studies [19]. In contrast, random-effects model attempted to generalize findings beyond the included studies by assuming that the selected studies are random samples from a larger population [20], and the stability of the results was verified by removing studies one by one. *P* values < 0.05 indicated statistically significant differences. Subgroups were used to analyze the effect of KT at different times after strenuous exercise. Funnel plots were used to assess publication bias when ≥10 studies were included.

If study outcomes were presented as median values, we contacted the author to obtain mean values. If standard error (SE) was provided instead of SD, the following formula was used to convert SE to SD: SD = SE × √(*n*), where *n* is the sample size [21]. If the mean and standard deviations as figures, we contacted the author to obtain actual values, and if no response is received, then the values are extracted using appropriate digitizing software.

## 3. Results

### 3.1. Search Results

**Figure 1:**
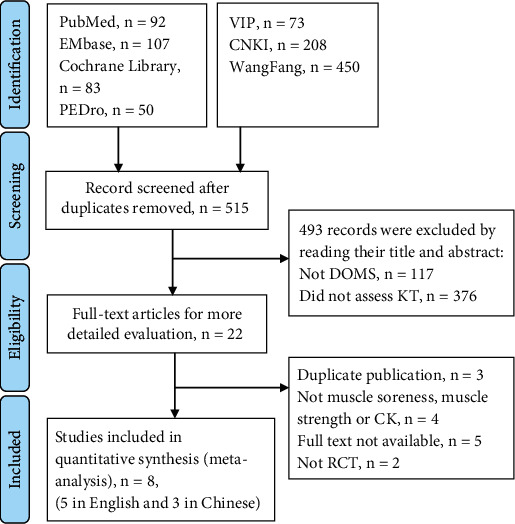
Flowchart of the included articles. VIP: Chinese Scientific Journal Database; CNKI: China National Knowledge Information database.

A total of 1,063 studies were identified via database searches. After removing duplicates, 515 studies remained for the initial screening, 493 of which were excluded after review of their titles and abstracts. After reviewing the full text of 22 studies, eight were found to meet the study inclusion criteria and were pooled for meta-analysis [4, 5, 22–27] (Figure 1).

### 3.2. Description of Included Studies

Eight studies involving a total of 289 participants from three Chinese [22, 26, 27] and five English articles [4, 5, 23–25] [4, 9, 11, 12, 28] were included in the final analyses. The included studies were conducted in China (*n* = 3, 37.5%), Thailand (*n* = 1, 12.5%), Turkey (*n* = 1, 12.5%), the Republic of Korea (*n* = 1, 12.5%), Cyprus (*n* = 1, 12.5%), and Poland (*n* = 1, 12.5%). Five studies included only male participants [23–26], a study included only female participants [4], and studies included both male and female participants [5, 22]. Most of the participants were in their 20s. The participants included athletes [20, 21, 23, 24], nonathlete volunteers [5], and healthy volunteers [4, 24–27]. Six studies assessed the quadriceps muscles [4, 5, 22, 23, 26, 27], one study assessed the biceps brachii muscle, and one study assessed both the quadriceps and gastrocnemius muscles [24, 25] (Table 1).

### 3.3. Risk of Bias Assessment

**Figure 2:**
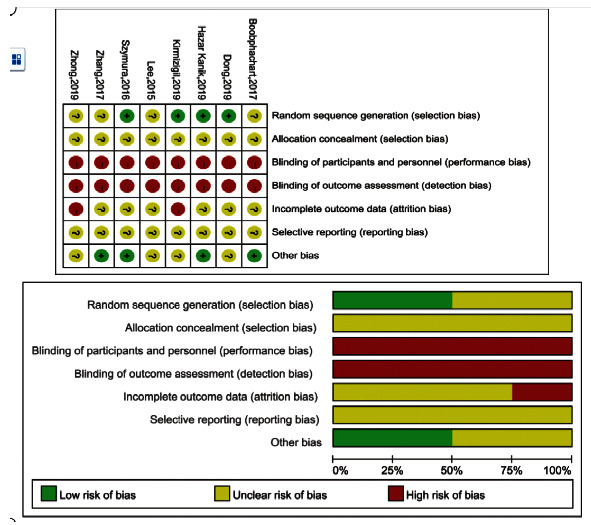
Risk of bias summary and graph.

All eight studies reported randomization, whereas four described the randomization method used to generate an allocation sequence [3, 9, 16, 19]. However, none of the studies mentioned allocation concealment. Participant blinding was inadequate in all eight studies. Although three studies reported single blinding using placebos as controls [4, 5, 22, 26], participants' previous experience with KT was not assessed. Blinding for outcome assessor was inadequate for all studies because they were able to see which participants had KT on them during the assessment. Two articles recorded dropouts [23, 26] but did not mention how missing values were treated in the data analysis, which increased the risk of attrition bias. The remaining studies did not report whether there were dropout cases. To determine selective reporting bias, we planned to assess these reports by investigating the study protocol. However, we were unable to complete this by search databases, and whether these eight studies had selective bias remains unclear (Figure 2).

### 3.4. Effects of KT on Muscle Soreness

**Figure 3:**
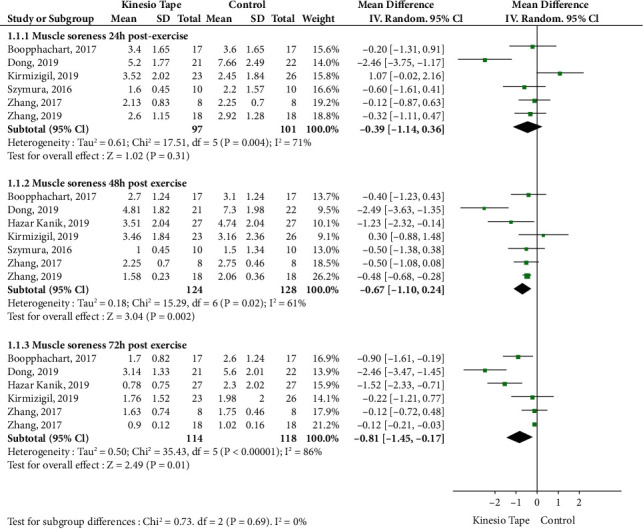
Meta-analysis of Kinesio tape on quadriceps muscle soreness.

Eight RCTs were analyzed for muscle soreness (seven RCTs for quadriceps muscle and one for biceps brachii muscle) at 24, 48, and 72 h postexercise. These studies included a total of 289 patients (142 in the KT groups and 147 in the control groups). For subgroup analysis of the quadriceps muscle, KT significantly decreased muscle soreness ratings at both 48 (MD: -0.67, 95% CI: -1.10 to 0.24, *P* = 0.002, *I*^2^ = 61%) and 72 h postexercise (MD: -0.81, 95% CI: -1.45 to -0.17, *P* = 0.01, *I*^2^ = 86%), compared with the control group. However, there was no significant difference at 24 h postexercise (MD: -0.39, 95% CI: -0.14 to 0.36, *P* = 0.31, *I*^2^ = 71%). A sensitivity analysis revealed relatively stable performance of the fixed- and random-effects models, which were also stable when trials were removed one by one (Figure 3). One RCT was analyzed for soreness in the biceps brachii muscle, which included 37 patients (18 in the KT group and 19 in the control group). The study indicated that KT use significantly decreased muscle soreness ratings compared with the control group at 72 h postexercise.

### 3.5. Effects of KT on Quadriceps Muscle Strength

**Figure 4:**
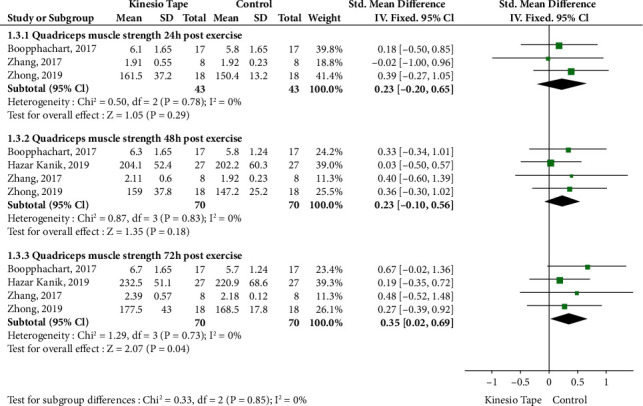
Meta-analysis of Kinesio tape on quadriceps muscle strength.

Four RCTs were analyzed, including 140 patients (70 in the KT group and 70 in the control group). For subgroup analysis, at 72 h postexercise, KT significantly increased quadriceps muscle strength compared with the control group (SMD: 0.35, 95% CI: 0.02 to 0.69, *P* = 0.04, *I*^2^ = 0%). However, there was no significant difference between subgroups at 24 (SMD: 0.23, 95% CI: -0.20 to 0.65, *P* = 0.29, *I*^2^ = 0%) or 48 h postexercise (SMD: 0.23, 95% CI: -0.10 to 0.56, *P* = 0.18, *I*^2^ = 0%) (Figure 4). A sensitivity analysis revealed relatively stable performance of the fixed- and random-effects models, which were also stable when trials were removed one by one.

### 3.6. Effects of KT on Serum CK Level

**Figure 5:**
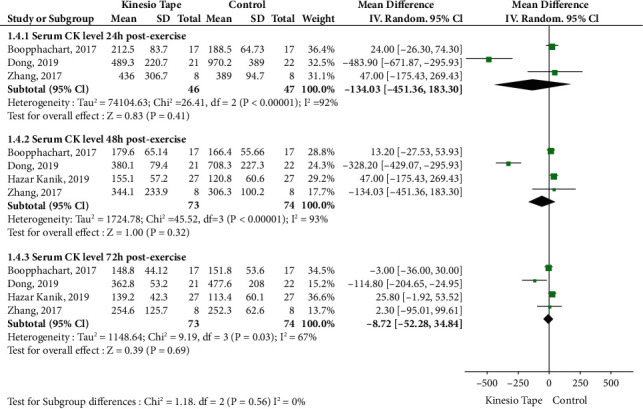
Meta-analysis of Kinesio tape on serum CK level. CK: creatine kinase.

Four RCTs were analyzed, including 147 patients (73 in the KT group and 74 in the control group). There was no significant difference in the subgroups at 24, 48, or 72 h postexercise compared to the control group (Figure 5). A sensitivity analysis revealed instability when a trial was removed.

## 4. Discussion

This systematic review evaluated eight RCTs, including 289 patients, and assessed the effect of KT on DOMS after strenuous exercise. Our analyses indicated that use of KT after strenuous exercise not only reduced muscle soreness but also improved muscle strength. However, KT use had no significant effect on postexercise CK levels.

These findings demonstrate that KT is more effective for pain relief at 48 and 72 h postexercise than at 24 h postexercise. This suggests that KT is better left on the skin for more than 48 hours postexercise to relieve the pain from DOMS. The potential explanations could be attributed to elevation of the skin, leading to an increase in subcutaneous space, which promotes circulation and lymphatic reflux, thereby eliminating the accumulation of painful substances and increasing muscle oxygenation. As KT changes the length of the muscle, proprioception, which is sensitive to the length of the muscle, is thought to be stimulated, resulting in muscle suppression; this phenomenon may also explain the effect of KT to reduce muscle soreness [28].

The effect of KT on muscle strength has been a research focus in sports medicine over the previous years. The present study revealed that KT significantly promotes recovery of muscle strength, especially at 72 h postexercise. KT may increase muscle strength by transmitting a pulling force that promotes alternate fascia movement [29]. However, KT also promotes muscle activity and thus improves muscle strength [30]. Muscle soreness limits muscle performance. However, KT may reduce muscle soreness, as demonstrated herein. However, whether KT improves muscle strength by reducing muscle pain is unclear and requires further research.

CK is considered as a biomarker of muscle injury [31, 32]. Muscle fiber damage causes CK in the muscles to concentrate in the blood [6]. A previous study has reported that elevated CK may indicate excessive exercise and DOMS, which leads to muscle tissue injury and edema [33]. Furthermore, because KT improves blood and lymph circulation to reduce edema [34, 35], it may also enhance damaged muscle repair. However, the results of the present study found no effect of KT on CK levels after strenuous exercise. There was no significant difference in CK levels with or without KT in a subgroup analysis at 24, 48, and 72 h postexercise. No differences in CK because of such few studies included for this outcome measure. Also, CK was known to exhibit substantially high interindividual variability. Nevertheless, results of a sensitivity analysis were not stable, and further analyses revealed different effects of KT on CK levels. For instance, three studies suggested that KT increased CK levels [4, 22, 26], whereas, conversely, another trial found that KT significantly reduced CK levels [5]. These opposing findings indicate that the impacts of KT use on CK levels are unstable and have a high degree of heterogeneity. Because of this high heterogeneity, the impact of KT on CK levels should be considered with caution, and further studies should be conducted to provide additional clarity.

The previous studies have several limitations based on the results of our critical appraisal. First, the methodological quality of the included studies was relatively low. None of the studies mentioned allocation concealment, although they did use randomization methods. Second, three articles used placebos to control for participant blinding, but previous experience of participants with KT was not assessed, whereas the other studies did not blind participants. Third, the number of studies included was limited, and most participants were in their 20s. Participant age may not be a significant limitation as DOMS is usually induced by excessive exercise in young athletes and sports enthusiasts [9].

The present meta-analysis has also several limitations. Even though a funnel plot was used to evaluate for publication bias among the included studies, the small number of studies was included, so conclusions about publication bias are limited. Furthermore, the language of the included studies was limited to English and Chinese, which may have led to some omissions. Future studies should therefore include a broader range of study languages. Finally, the wide variation in methodological designs (i.e., participant and control intervention) contributed to a high degree of heterogeneity in muscle soreness outcomes, although the total outcomes were relatively stable. A sensitivity analysis revealed that the CK outcome was not stable; thus, these results should be considered with caution. Well-designed RCTs are required in the future to better evaluate the effect of KT on CK. Studies ascertaining changes in CK levels in the development of delayed muscle soreness are also needed.

This systematic review and meta-analysis revealed that KT effectively treats the symptoms of DOMS with a maximum efficacy at 72 h postexercise. KT also increases muscle strength at 72 h postexercise. Given these findings, KT may be a useful method for the alleviation of DOMS. However, considering the relatively poor methodological quality of the studies included, the results reported here should be interpreted with caution. Additionally, large-sized, randomized, blinded trials are needed to clarify the actual role of KT in patients with DOMS.

## Figures and Tables

**Table 1:** Characteristics of the included studies.

First author, year	Age (years), mean ± SD	Method that induced DOMS	Assessed muscle	Sample size	KT^∗^ technique	Outcome	Time point
Boobphachart, 2017 [4]	KTG^§^: 43.9 ± 1.4	Eccentric exercise: 4 sets of 25 maximal voluntary eccentric contractions at a velocity of 60°/s with 3 min rest between sets	Quadriceps muscles	KT (*n* = 17)	Elastic tape (Kinesio® Tex Gold™) with 5 cm width and 20 cm length was applied on the rectus femoris	VAS^†^, peak torque, CK^¶^	0, 24, 48, and 72 h after DOMS‡
	CG^§^:43.1 ± 2.0			Placebo (*n* = 17)			

Dong, 2019 [22]	KTG: 19.1 ± 2.2	A knee extensor training instrument (Hur, Finland) was adopted, and 70% maximum load intensity (1 RM) was selected for knee stretching exercises (knee motion was 80°, 5 groups, 10 times/group, with 2-min rest between sets)	Quadriceps muscles	KT (*n* = 21)	The tape was cut in an I-shape, and Kinesio tape involved the quadriceps muscle	VAS, CK	0, 24, 48, and 72 h after DOMS
	CG: 18.8 ± 2.9			No treatment (*n* = 22)			

Hazar Kanik, 2019 [5]	22 (20–28) ||	Eccentric exercise: five sets of 20 drop jumps were performed with a 10 s interval between each jump and 2 min rest between sets	Quadriceps muscles	KT (*n* = 27)	The tape could be stretched to 140% of its original length, and the tape was left on the participants' skin for the next 72 h	VAS, maximal isometric muscle strength, CK	0, 48, and 72 h after DOMS
				Placebo (*n* = 27)			

Kirmizigil, 2019 [23]	21.36 ± 1.68	Eccentric exercise: 5 sets of 20 drop jumps from a 0.6-m-high box with 10 s intervals between each jump and 2 min rest between sets	Quadriceps muscles	KT (*n* = 23)	The 5 cm width KT (Kinesio® Tex Gold™) applied to the participants could be stretched to 140% of its original length; the tape was cut in a Y-shape and worn over a period of 3–5 days	VAS	0, 0.5, 24, 48, and 72 h after DOMS
				No treatment (*n* = 26)			

Lee, 2015 [24]	KTG: 22.5 ± 1.4	Eccentric exercise: dumbbells were used to elicit 70% of the maximal isometric muscle strength (1 RM) of each subject in order to induce DOMS; the participants slowly extended their elbows to the fully extended position against resistance	Biceps brachii muscle	KT (*n* = 19)	KT was applied to the biceps brachii on the nondominant side in a direction perpendicular to the muscle fibers	VAS	24, 48, and 72 h after DOMS
	CG: 23.5 ± 1.2			No treatment (*n* = 18)			
Szymura, 2016 [25]	21.2 ± 0.42	Eccentric exercise: two 60 min downhill runs with a constant intensity	Quadriceps and gastrocnemius muscles	KT (*n* = 10)	For lymphatic application of the Kinesio tape, a 5 cm width K-active tape was used for 48 h	VAS	0, 24, 48, 72 h after DOMS
				No treatment (*n* = 10)			

Zhang, 2017 [26]	KTG: 21.0 ± 1.1	Eccentric exercise: full-squat frog jump and 30 times half-squat jump with 10 kg load	Quadriceps muscles	KT (*n* = 8)	The 5 cm width KT applied to the participants had a feature of being stretched to 110% of its original length; Kinesio tape involved the quadriceps muscle over 4 days	VAS, peak torque, CK	0, 24, 48, and 72 h after DOMS
	CG: 20.6 ± 1.3			Placebo (*n* = 8)			

Zhong, 2019 [27]	KTG: 19.2 ± 0.8	Eccentric exercise: 15 times full-squat frog jump and 30 times half-squat jump with load, a total of 10 sets, with 2 min between sets to rest	Quadriceps muscles	KT (*n* = 18)	The 5 cm width KT applied to the participants can be stretched to 110% of its original length; Kinesio tape involved the quadriceps muscle over 4 days.	VAS, peak torque	0, 24, 48, and 72 h after DOMS
	CG: 19.0 ± 1.2			No treatment (*n* = 18)			

^§^KTG: Kinesio tape group; ^§^CG: control group; ^∗^KT: Kinesio tape; ^†^VAS: visual analogue scale; ^¶^CK: creatine kinase; ^**‡**^DOMS: delayed onset muscle soreness; ^||^median (minimum–maximum).

## Data Availability

The original data presented in the study are included in the article or supplementary material. Further inquiries can be directed to the corresponding authors.

## Supplementary Materials

Supplementary 1 Supplementary Data S1: details of search strategies (PDF).

Supplementary 2 Supplementary Data S2: PRISMA checklist (PDF).

## References

[1] Pedersen B. K., Saltin B. (2015). Exercise as medicine - evidence for prescribing exercise as therapy in 26 different chronic diseases. *Scandinavian Journal of Medicine & Science in Sports*.

[2] Barreto R. V., de Lima L. C. R., Greco C. C., Denadai B. S. (2019). Protective effect conferred by isometric preconditioning against slow- and fast-velocity eccentric exercise-induced muscle damage. *Frontiers in Physiology*.

[3] McGrath R. P., Whitehead J. R., Caine D. J. (2014). The effects of proprioceptive neuromuscular facilitation stretching on post-exercise delayed onset muscle soreness in young adults. *International Journal of Exercise Science*.

[4] Boobphachart D., Manimmanakorn N., Manimmanakorn A., Thuwakum W., Hamlin M. J. (2017). Effects of elastic taping, non-elastic taping, and static stretching on recovery after intensive eccentric exercise. *Research in Sports Medicine*.

[5] Kanik Z. H., Citaker S., Demirtas C. Y., Bukan N. C., Celik B., Gunaydin G. (2019). Effects of Kinesio taping on the relief of delayed onset muscle soreness: a randomized, placebo-controlled trial. *Journal of Sport Rehabilitation*.

[6] Cheung K., Hume P. A., Maxwell L. (2003). Delayed onset muscle soreness. *Sports Medicine*.

[7] Doma K., Deakin G. B., Bentley D. J. (2017). Implications of impaired endurance performance following single bouts of resistance training: an alternate concurrent training perspective. *Sports Medicine*.

[8] Doma K., Deakin G. B., Schumann M., Bentley D. J. (2019). Training considerations for optimising endurance development: an alternate concurrent training perspective. *Sports Medicine*.

[9] Ozmen T., Aydogmus M., Dogan H., Acar D., Zoroglu T., Willems M. (2016). The effect of Kinesio taping on muscle pain, sprint performance, and flexibility in recovery from squat exercise in young adult women. *Journal of Sport Rehabilitation*.

[10] Hyldahl R. D., Hubal M. J. (2014). Lengthening our perspective: morphological, cellular, and molecular responses to eccentric exercise. *Muscle & Nerve*.

[11] Fratocchi G., Di Mattia F., Rossi R., Mangone M., Santilli V., Paoloni M. (2013). Influence of Kinesio taping applied over biceps brachii on isokinetic elbow peak torque. A placebo-controlled study in a population of young healthy subjects. *Journal of Science and Medicine in Sport*.

[12] Huang C. Y., Hsieh T. H., Lu S. C., Su F. C. (2011). Effect of the Kinesio tape to muscle activity and vertical jump performance in healthy inactive people. *Biomedical Engineering Online*.

[13] Lemos T. V., de Souza Júnior J. R., Dos Santos M. G., Rosa M. M., da Silva L. G., Matheus J. P. (2018). Kinesio taping effects with different directions and tensions on strength and range of movement of the knee: a randomized controlled trial. *Brazilian Journal of Physical Therapy*.

[14] Fu T.-C., Wong A. M. K., Pei Y.-C., Wu K. P., Chou S.-W., Lin Y.-C. (2008). Effect of Kinesio taping on muscle strength in athletes-a pilot study. *Journal of Science and Medicine in Sport*.

[15] Vercelli S., Sartorio F., Foti C. (2012). Immediate effects of Kinesio taping on quadriceps muscle strength. *Clinical Journal of Sport Medicine*.

[16] de Almeida Lins C. A., Neto F. L., de Amorim A. B. C., de Brito Macedo L., Brasileiro J. S. (2013). Kinesio Taping (®) does not alter neuromuscular performance of femoral quadriceps or lower limb function in healthy subjects: randomized, blind, controlled, clinical trial. *Manual Therapy*.

[17] Moher D., Liberati A., Tetzlaff J., Altman D. G., PRISMA Group (2010). Preferred reporting items for systematic reviews and meta-analyses: the PRISMA statement. *International Journal of Surgery*.

[18] Higgins J. P., Thompson S. G., Deeks J. J., Altman D. G. (2003). Measuring inconsistency in meta-analyses. *BMJ*.

[19] Cheung M. W., Ho R. C., Lim Y., Mak A. (2012). Conducting a meta-analysis: basics and good practices. *International Journal of Rheumatic Diseases*.

[20] Lim R., Zhang M., Ho R. (2018). Prevalence of all-cause mortality and suicide among bariatric surgery cohorts: a meta-analysis. *International Journal of Environmental Research and Public Health*.

[21] Higgins J. P. T., Altman D. G., Gotzsche P. C. (2011). The Cochrane collaboration’s tool for assessing risk of bias in randomised trials. *BMJ*.

[22] Dong Q. Z. (2019). Effect of kinesio taping on delayed onset muscle soreness in athletes. *Chinese Journal of Tissue Engineering Research*.

[23] Kirmizigil B., Chauchat J. R., Yalciner O., Iyigun G., Angin E., Baltaci G. (2019). The effectiveness of Kinesio taping in recovering from delayed onset muscle soreness: a cross-over study. *Journal of Sport Rehabilitation*.

[24] Lee Y. S., Bae S. H., Hwang J. A., Kim K. Y. (2015). The effects of Kinesio taping on architecture, strength, and pain of muscles in delayed onset muscle soreness of biceps brachii. *Journal of physical therapy science*.

[25] Szymura J., Maciejczyk M., Wiecek M. (2016). Effects of Kinesio taping on anaerobic power recovery after eccentric exercise. *Research in Sports Medicine*.

[26] Zhang G. H., Wang R. W. (2017). The effects of kinesio taping on delayed onset muscle soreness and recovery of muscle function. *China Sport Science*.

[27] Zhong G. Y. (2019). Vibration combined with kinesio taping treats delayed-onset muscle soreness. *Chinese Journal of Tissue Engineering Research*.

[28] Kim J., Kim J., Lee J. (2017). Effect of compression garments on delayed-onset muscle soreness and blood inflammatory markers after eccentric exercise: a randomized controlled trial. *Journal of Exercise Rehabilitation*.

[29] Yam M. L., Yang Z., Zee B. C.-Y., Chong K. C. (2019). Effects of Kinesio tape on lower limb muscle strength, hop test, and vertical jump performances: a meta-analysis. *BMC Musculoskeletal Disorders*.

[30] Hsu Y. H., Chen W. Y., Lin H. C., Wang W. T. J., Shih Y. F. (2009). The effects of taping on scapular kinematics and muscle performance in baseball players with shoulder impingement syndrome. *Journal of Electromyography and Kinesiology*.

[31] Brancaccio P., Maffulli N., Limongelli F. M. (2007). Creatine kinase monitoring in sport medicine. *British Medical Bulletin*.

[32] Tojima M., Noma K., Torii S. (2016). Changes in serum creatine kinase, leg muscle tightness, and delayed onset muscle soreness after a full marathon race. *The Journal of Sports Medicine and Physical Fitness*.

[33] Tee J. C., Bosch A. N., Lambert M. I. (2007). Metabolic consequences of exercise-induced muscle damage. *Sports Medicine*.

[34] da Rocha Heras A. C. T., de Oliveira D. M. S., Guskuma M. H. (2020). Kinesio taping use to reduce pain and edema after third molar extraction surgery: a randomized controlled split-mouth study. *Journal of Cranio-Maxillofacial Surgery*.

[35] Windisch C., Brodt S., Röhner E., Matziolis G. (2017). Effects of Kinesio taping compared to arterio-venous Impulse System™ on limb swelling and skin temperature after total knee arthroplasty. *International Orthopaedics*.

