# Supplementary material for: Effects of Kinesio Tape on Delayed Onset Muscle Soreness: A Systematic Review and Meta-analysis
Source: Biomed Res Int. 2021 May 31;2021:6692828. doi: 10.1155/2021/6692828 (PMC8188598; doi:10.1155/2021/6692828)
Supplement: Supplementary 1 — Supplementary Data S1: details of search strategies (PDF). [file 6692828.f1.pdf]

Database	Search Strategy	Result
**Pubmed, N=92**	((((("athletic tape"[MeSH Terms] OR ("athletic"[All Fields] AND "tape"[All Fields]) OR "athletic tape"[All Fields] OR ("tape"[All Fields] AND "athletic"[All Fields])) OR ("athletic tape"[MeSH Terms] OR ("athletic"[All Fields] AND "tape"[All Fields]) OR "athletic tape"[All Fields] OR ("orthotic"[All Fields] AND "tape"[All Fields]) OR "orthotic tape"[All Fields])) OR ("athletic tape"[MeSH Terms] OR ("athletic"[All Fields] AND "tape"[All Fields]) OR "athletic tape"[All Fields] OR ("kinesio"[All Fields] AND "tape"[All Fields]) OR "kinesio tape"[All Fields])) OR ("athletic tape"[MeSH Terms] OR ("athletic"[All Fields] AND "tape"[All Fields]) OR "athletic tape"[All Fields] OR "kinesiotape"[All Fields])) AND ("muscles"[MeSH Terms] OR "muscles"[All Fields] OR "muscle"[All Fields])) AND ("exercise"[MeSH Terms] OR "exercise"[All Fields])	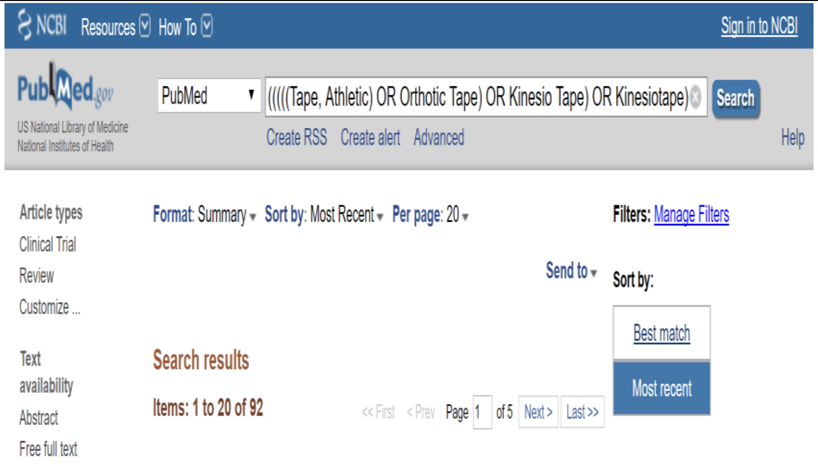

**Cochrane library, N=83**	Kinesio tape and muscle	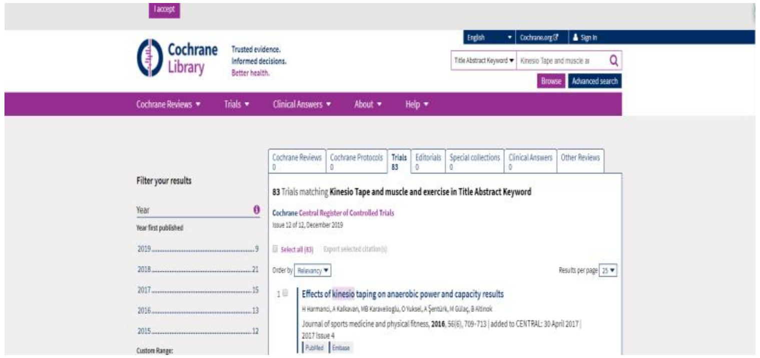
**PEDro, N=50**	Kinesio tape and muscle	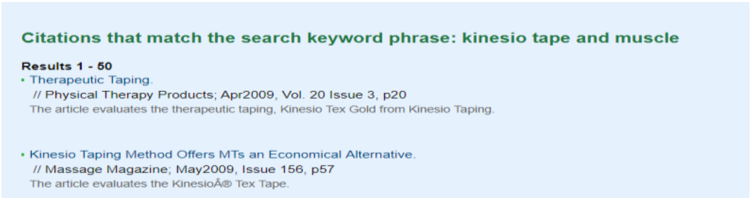
**Embase. N=107**	(kinesio AND tape OR (tape, AND athletic) OR (tape, AND orthotic)) AND muscle AND ('exercise'/exp OR exercise)	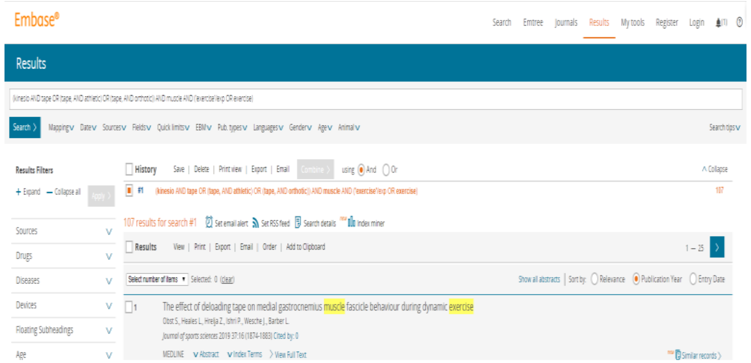

**CNKI, N=208**	Kinesio tape and muscle and exercise	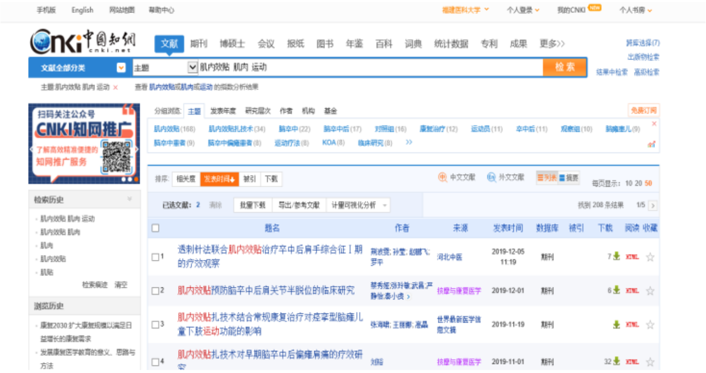
**VIP, N=73**	Kinesio tape and muscle	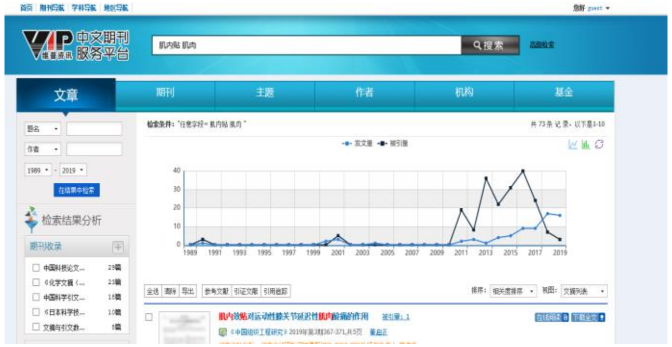

**WangFang, N=450**	Kinesio tape and muscle and exercise	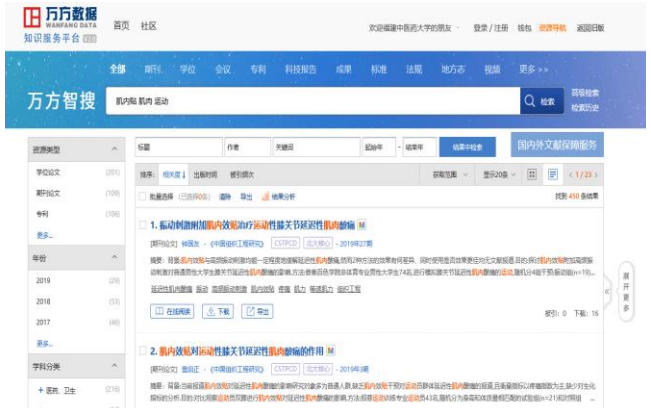

S1. Details of search strategies (Electronic search strategy and result for seven database.)
