# Supplementary material for: Effects of Kinesio Tape on Delayed Onset Muscle Soreness: A Systematic Review and Meta-analysis
Source: Biomed Res Int. 2021 May 31;2021:6692828. doi: 10.1155/2021/6692828 (PMC8188598; doi:10.1155/2021/6692828)
Supplement: Supplementary 2 — Supplementary Data S2: PRISMA checklist (PDF). [file 6692828.f2.docx]

| **Checklist of items to include when reporting a systematic review or meta-analysis** | | | |
| --- | --- | --- | --- |
| **Section/Topic** | **Item** | **Checklist Item** | **Reported on page** |
| **TITLE** |  |  |  |
| Title | 1 | Identify the report as a systematic review, meta-analysis, or both. | **page:** 1 |
| **ABSTRACT** |  |  |  |
| Structured summary | 2 | Provide a structured summary including, as applicable: background; objectives; data sources; study eligibility criteria, participants, and interventions; study appraisal and synthesis methods; results; limitations; conclusions and implications of key ﬁndings; systematic review registration number. | **page** :1 |
| **INTRODUCTION** |  |  |  |
| Rationale | 3 | Describe the rationale for the review in the context of what is already known. | **page** :2-3 |
| Objectives | 4 | Provide an explicit statement of questions being addressed with reference to participants, interventions, comparisons, outcomes, and study design (PICOS). | **page** :3 |
| **METHODS** |  |  |  |
| Protocol and registration | 5 | Indicate if a review protocol exists, if and where it can be accessed (eg, Web address), and, if available, provide registration information including registration number. | **page** :3-4 |
| Eligibility criteria | 6 | Specify study characteristics (eg, PICOS, length of follow-up) and report characteristics (eg, years considered, language, publication status) used as criteria for eligibility, giving rationale. | **page** :4 |
| Information sources | 7 | Describe all information sources (eg, databases with dates of coverage, contact with study authors to identify additional studies) in the search and date last searched. | **page** :3-4 |
| Search | 8 | Present full electronic search strategy for at least one database, including any limits used, such that it could be repeated. | **page** :3-4 |
| Study selection | 9 | State the process for selecting studies (ie, screening, eligibility, included in systematic review, and, if applicable, included in the meta-analysis). | **page** :4 |
| Data collection process | 10 | Describe method of data extraction from reports (eg, piloted forms, independently, in duplicate) and any processes for obtaining and conﬁrming data from investigators. | **page** :5 |
| Data items | 11 | List and deﬁne all variables for which data were sought (eg, PICOS, funding sources) and any assumptions and simpliﬁcations made. | **page** : 5 |
| Risk of bias in individual studies | 12 | Describe methods used for assessing risk of bias of individual studies (including speciﬁcation of whether this was done at the study or outcome level), and how this information is to be used in any data synthesis. | **page** :4-5 |
| Summary measures | 13 | State the principal summary measures (eg, risk ratio, difference in means). | **page** :5-6 |
| Synthesis of results | 14 | Describe the methods of handling data and combining results of studies, if done, including measures of consistency (eg, I^2^) for each meta-analysis. | **page** :5-6 |
| Risk of bias across studies | 15 | Specify any assessment of risk of bias that may affect the cumulative evidence (eg, publication bias, selective reporting within studies). | **page** :5-6 |
| Additional analyses | 16 | Describe methods of additional analyses (eg, sensitivity or subgroup analyses, meta-regression), if done, indicating which were pre-speciﬁed. | **page** :5-6 |
| **RESULTS** |  |  |  |
| Study selection | 17 | Give numbers of studies screened, assessed for eligibility, and included in the review, with reasons for exclusions at each stage, ideally with a ﬂow diagram. | **page** :6 |
| Study characteristics | 18 | For each study, present characteristics for which data were extracted (eg, study size, PICOS, follow-up period) and provide the citations. | **page** :6-7 |
| Risk of bias within studies | 19 | Present data on risk of bias of each study and, if available, any outcome-level assessment (see Item 12). | **page** :7 |
| Results of individual studies | 20 | For all outcomes considered (beneﬁts or harms), present, for each study: (a) simple summary data for each intervention group and (b) effect estimates and conﬁdence intervals, ideally with a forest plot. | **page** :7-9 |
| Synthesis of results | 21 | Present results of each meta-analysis done, including conﬁdence intervals and measures of consistency. | **page** : 7-9 |
| Risk of bias across studies | 22 | Present results of any assessment of risk of bias across studies (see Item 15). | **page** : 7-9 |
| Additional analysis | 23 | Give results of additional analyses, if done(eg, sensitivity or subgroup analyses, meta-regression[see Item 16]). | **page** :7-9 |
| **DISCUSSION** |  |  |  |
| Summary of evidence | 24 | Summarize the main ﬁndings including the strength of evidence for each main outcome; consider their relevance to key groups (eg, health care providers, users, and policy makers). | **page** :9-10 |
| Limitations | 25 | Discuss limitations at study and outcome level (eg, risk of bias), and at review level (eg, incomplete retrieval of identiﬁed research, reporting bias). | **page** :10 |
| Conclusions | 26 | Provide a general interpretation of the results in the context of other evidence, and implications for future research. | **page** :11 |
| **FUNDING** |  |  |  |
| Funding | 27 | Describe sources of funding for the systematic review and other support (eg, supply of data); role of funders for the systematic review. | **page** :11 |
